# Supplementary material for: Proposal for a physiotherapy assessment form for the evaluation of women patients with uro-gynecological disorders: A Delphi study
Source: PLoS One. 2023 Dec 27;18(12):e0296092. doi: 10.1371/journal.pone.0296092 (PMC10752553; doi:10.1371/journal.pone.0296092)
Supplement: Attachment — Submitted filename: Dear Reviewers and Editor of PLOS One.docx [file pone.0296092.s001.docx]

Dear Reviewers and Editor of PLOS One:

Thank you very much for your suggestions and contributions to improve the quality of the manuscript. Following your indications, we respond, point by point, to the reviewers' comments.

In the text, all the modified or added sentences have been written in red to facilitate the correction by the reviewers.

**Editor comments:**

1. Thank you for providing your data set using a repository. If you could please translate this data into English as per PLOS Data Regulations we ask for data to be ‘fully available without restriction’.

*The Authors have published in Zenodo the data related to the two rounds of the Delphi study carried out through two Excel files fully translated into English.*

Once again, thank you very much for the time spent and the interest shown in this work; as well as in the positive evaluations you have given of it.

Receive a warm greeting,

The authors.
